# Recovery of ^177^Lu from Irradiated HfO_2_ Targets for Nuclear Medicine Purposes

**DOI:** 10.3390/molecules27103179

**Published:** 2022-05-16

**Authors:** Andrey G. Kazakov, Taisya Y. Ekatova, Julia S. Babenya, Sergey S. Belyshev, Vadim V. Khankin, Alexander A. Kuznetsov, Sergey E. Vinokurov, Boris F. Myasoedov

**Affiliations:** 1 Radiochemistry Laboratory, Vernadsky Institute of Geochemistry and Analytical Chemistry of the Russian Academy of Sciences (GEOKHI RAS), Kosygin St., 19, 119991 Moscow, Russia; ekatova.t@gmail.com (T.Y.E.); julia.babenya@gmail.com (J.S.B.); vinokurov.geokhi@gmail.com (S.E.V.); bfmyas@mail.ru (B.F.M.); 2 Skobeltsyn Institute of Nuclear Physics, Lomonosov Moscow State University, Leninskie Gory, 1, Bld. 2, 119991 Moscow, Russia; belyshev@depni.sinp.msu.ru (S.S.B.); v-k32@yandex.ru (V.V.K.); kuznets@depni.sinp.msu.ru (A.A.K.); 3 Department of Physics, Lomonosov Moscow State University, Leninskie Gory, 1, Bld. 2, 119991 Moscow, Russia

**Keywords:** ^177^Lu, extraction chromatography, photonuclear reactions, radiochemical recovery, nuclear medicine

## Abstract

A new method of production of one of the most widely used isotopes in nuclear medicine, ^177^Lu, with high chemical purity was developed; this method includes irradiation of the HfO_2_ target with bremsstrahlung photons. The irradiated target was dissolved in HF and then diluted and placed onto a column filled with LN resin. Quantitative sorption of ^177^Lu could be observed during this process. The column later was rinsed with the mixture of 0.1 M HF and 1 M HNO_3_ and then 2 M HNO_3_ to remove impurities. Quantitative desorption of ^177^Lu was achieved by using 6 M HNO_3_. The developed method of ^177^Lu production ensures high purification of this isotope from macroquantities of hafnium and zirconium and radioactive impurities of carrier-free yttrium. The content of ^177m^Lu in ^177^Lu in photonuclear production was determined. Due to high chemical and radionuclide purity, ^177^Lu obtained by the developed method can be used in nuclear medicine.

## 1. Introduction

^177^Lu is one of the most known and widely used therapeutic radioisotopes in nuclear medicine [1]. The first clinical application of ^177^Lu happened in the 1960s; however, the breakthrough in using radiopharmaceuticals based on this radionuclide occurred later with the development of the ^177^Lu-DOTATATE complex designed to treat neuroendocrine tumors [2,3,4]. Nowadays, the main method of ^177^Lu production is the irradiation of ^176^Lu or ^176^Yb in nuclear reactors, and this production has its disadvantages. Thus, the operation of reactors leads to the accumulation of nuclear waste; irradiation of ^176^Lu leads to ^177^Lu with carrier; recovery of ^177^Lu from neighbor lanthanide Yb is not a simple task [1]. As a result, other perspective methods of ^177^Lu production have been investigated recently, the photonuclear one in particular. Today, this method is used for medical isotope production. For instance, light isotopes ^11^C, ^13^N, ^15^O, and ^18^F as well as radiometals ^47^Sc, ^67^Cu, and ^99^Mo/^99m^Tc generators are regularly obtained in sufficient quantities using electron accelerators [5]. Compared to the use of nuclear reactors, the photonuclear method has the following advantages: the compact sizes of electron accelerators that create an opportunity of placing one near the hospital and the relatively cheap cost of accelerators’ functioning. The main disadvantage is cross-sections of photonuclear reactions that are usually lower than ones in reactor production. Data about photonuclear production of ^177^Lu from hafnium are limited [6,7,8].

One of the issues of ^177^Lu production is the formation of long-lived isomer ^177m^Lu (T_1/2_ = 160.4 d), the content of which should be minimized in ^177^Lu-based radiopharmaceuticals (the activities ratio of ^177m^Lu and ^177^Lu should not exceed 0.02 %). The method of ^177^Lu recovery from irradiated HfO_2_ using extraction chromatography was developed by us to determine the amount of formed ^177m^Lu in purified lutetium fractions [8]; any other techniques of recovery of trace amounts of lutetium from macroquantities of hafnium are absent. However, no gamma-ray peaks of ^177m^Lu were observed during a long registration of the gamma-ray spectrum of purified lutetium solution due to the registration of yttrium isotopes forming from zirconium impurities contained in the initial sample of HfO_2_; their Compton plateau in the gamma-ray spectrum overlaps with ^177m^Lu peaks. Consequently, the development of methods of additional purification of recovered ^177^Lu from forming during irradiation yttrium isotopes is a task of current interest.

The isotope ^177^Lu can be produced either from hafnium with natural isotopic composition or from enriched ^178^Hf, which is notably more expensive. It was mentioned that to produce ^177^Lu with radionuclide purity sufficient for nuclear medicine, it is necessary to irradiate massive (a few grams) targets made of ^178^Hf [8] and, as a result, such expensive target material should be reused after the recovery of ^177^Lu.

The purpose of this work was to develop a promising method of ^177^Lu production from HfO_2_ using electron accelerator with subsequent purification from hafnium, zirconium, and especially yttrium, to develop a technique of irradiated HfO_2_ regeneration, and also to determine the amount of ^177m^Lu produced during this irradiation. 

## 2. Results and Discussion

**Figure 1:**
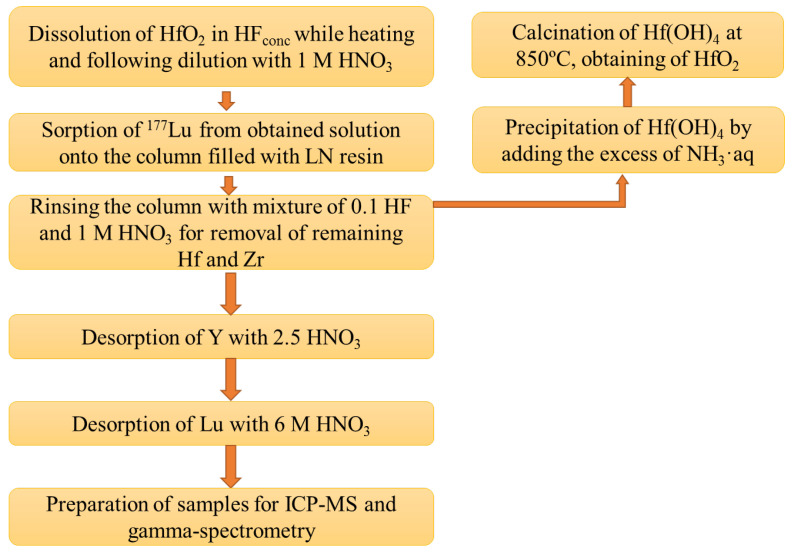
Scheme of developed method of ^177^Lu recovery after its production in electron accelerators and regeneration of HfO_2_.

In our work, a new method of ^177^Lu production for nuclear medicine was developed: irradiation of HfO_2_ with bremsstrahlung photons, its dissolution, and recovery of ^177^Lu using extraction chromatography; the corresponding scheme is presented in Figure 1.

### 2.1. Development of Method of Recovery of ^177^Lu from Macroquantities of Hafnium and Zirconium, Trace Amounts of Yttrium Isotopes

**Figure 2:**
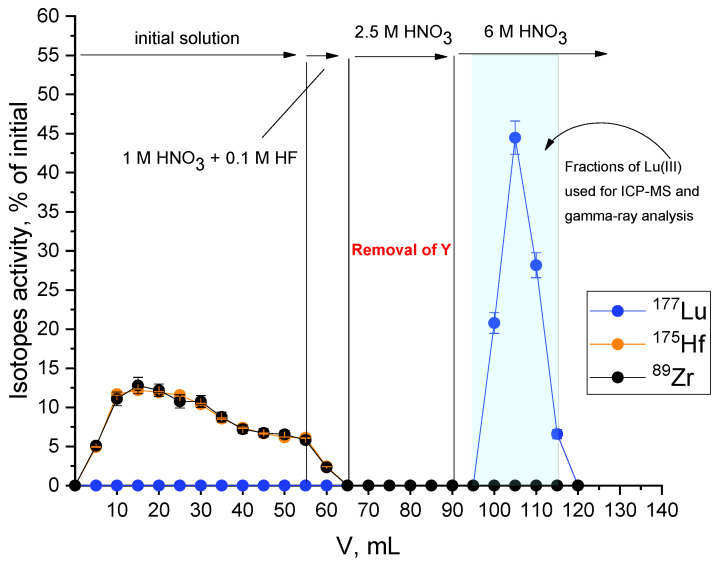
Elution curves of Hf, Zr, and Lu in developed recovery method. Activities of Y isotopes were too low to make its elution curve.

Method of ^177^Lu recovery from irradiated HfO_2_, developed previously by us, included the following steps: target material dissolution in HF_conc_ and dilution of the obtained solution fifteen times with 1 M HNO_3_; sorption of lutetium on the column filled with LN resin (based on di(2-ethylhexyl)orthophosporic acid); rinsing the column with 1 M HNO_3_ and 0.1 M HF mixture and then with 1 M HNO_3_ for the remaining hafnium and for fluoride ion removals, accordingly; and, finally, rinsing with 6 M HNO_3_ to desorb ^177^Lu [8]. To further purify ^177^Lu from yttrium, the step of column rinsing with 2.5 M HNO_3_ was introduced before the desorption of ^177^Lu; this solution was proved to be the optimal media for quantitative desorption of yttrium according to the conducted experiments (see Supplementary Section). Table 1 contains data on the content of hafnium, zirconium, yttrium, and lutetium in eluates obtained during different stages of the developing technique; a chromatogram is presented in Figure 2.

It was established that hafnium and zirconium did not sorb onto the column during the sorption of lutetium; however, approximately 60% of yttrium was sorbed. Yttrium quantitatively desorbed during the column rinsing with 2.5 M HNO_3_, while lutetium remained on the column. During the subsequent rinsing with 6 M HNO_3_, ^177^Lu quantitatively (no less than 98%) desorbed from the column. 

Hafnium content in the obtained solution of lutetium was lower than the detection limit of ICP-MS. This led to the conclusion that the real content of hafnium in the ^177^Lu solution during the recovery following this method is 1.2 × 10^10^ times lower compared to the content in the initial solution, which is five orders of magnitude higher than the result obtained by us earlier [8]. Zirconium content in the final product, according to ICP-MS, is 1.7 × 10^6^ times lower than in the initial solution. As for the purification of lutetium from yttrium, no peaks of yttrium isotopes were detected in a gamma-ray spectrum of lutetium solution aliquot during prolonged registration. Thus, according to the detection limit of ^88^Y, yttrium content in the final solution was 10^4^ times lower than in the initial one.

### 2.2. Recovery of ^177^Lu from Irradiated HfO_2_, Determination of ^177m^Lu Content, and Regeneration of HfO_2_

A target with a mass of 16 g was irradiated with bremsstrahlung photons with energy up to 55 MeV for 8 h; then, it was dissolved in HF, and the recovery was conducted according to the technique described above. It was established that the purification degree was achieved as outlined above, and the lutetium yield was 98.5 ± 0.5%.

**Figure 3:**
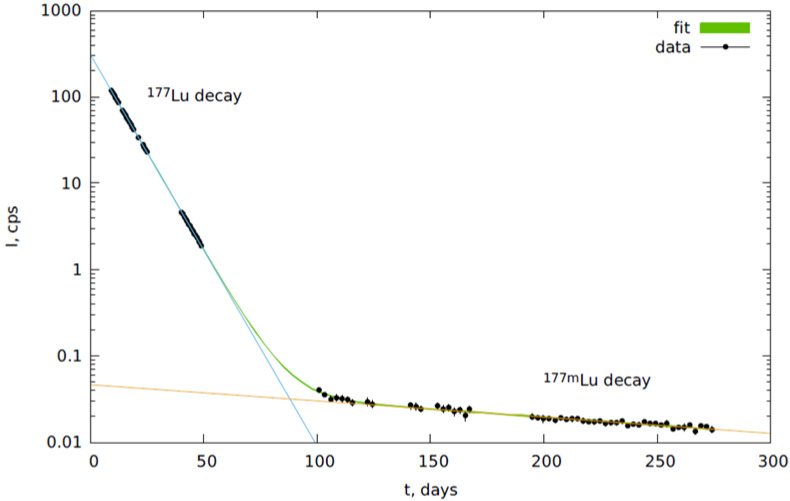
Dependency of count rate of 208 keV line, which is present in both ^177^Lu and ^177m^Lu spectra, on time after irradiation. Dots—experimental data, lines—fit. Blue lines—fit of ^177^Lu decay, orange lines—fit of ^177m^Lu decay.

The high purification level of lutetium from macroquantities of hafnium, zirconium, and trace amounts of yttrium, formed during the irradiation of zirconium achieved in our work, allowed us to detect ^177m^Lu peaks during prolonged registration of the gamma-ray spectrum of the obtained lutetium. Figure 3 presents the dependency of count rate of the 208 keV line, which is the most intense for both ^177^Lu and ^177m^Lu, on time after the lutetium isotope’s recovery. It can be seen in Figure 1 that it is possible to determine the contribution of ^177m^Lu in the count rate of this line after ^177^Lu decay, which allows us to precisely determine the radioactivity of ^177m^Lu after the irradiation. Thus, the ratio of the activity of ^177m^Lu to the activity of ^177^Lu in the photonuclear production of ^177^Lu was established to be (2.87 ± 0.07) × 10^−5^ (or 0.00287%). Table 2 allows us to compare the activity ratios ^177m^Lu/^177^Lu in production by different methods, and it is clear that the ratio in case of the photonuclear method is minimal among direct production routes, and ^177^Lu obtained by this method can be used in nuclear medicine. 

According to X-ray diffraction (XRD), the spectra of commercial HfO_2_ and the product of calcination of hafnium hydroxide obtained during the recovery of ^177^Lu are identical, and values of interplanar distances coincide with the values for HfO_2_ from the database. Thus, after heating, HfO_2_ can be stored to decrease activity of hafnium isotopes if necessary, and can be reused for irradiation for ^177^Lu production.

### 2.3. Comparison of Methods of Obtaining Carrier-Free ^177^Lu

We demonstrated the possibility of producing and separating ^177^Lu for nuclear medicine using electron accelerators. In conclusion, we present a comparison of this method and the production of ^177^Lu without a carrier in a reactor and a cyclotron.

In the case of ^176^Yb irradiation in the reactor, it is possible to produce 1.8 GBq of ^177^Lu (therapeutic activity) by irradiating 5 mg of 97.6% ^176^Yb_2_O_3_ for 10 days using flux of 1 × 10^14^ n∙cm^−2^∙s^−1^ [12]. According to calculations based on experimental data, the same activity of ^177^Lu can be obtained by irradiating a 100 µm plate of 100% ^176^Yb with deuterons for about 2 h at a current of 0.1 mA [13]. According to our earlier theoretical calculations, 1.8 GBq of carrier-free ^177^Lu can be produced in an electron accelerator by irradiating an enriched ^179^HfO_2_ target at a current of 0.1 mA [8]. However, it is important to note that the results of calculating the yields of photoproton reactions are usually underestimated from several times to several orders of magnitude. Thus, the determination of the experimental values of ^177^Lu yields upon irradiation of enriched targets made of ^178^Hf or ^179^Hf is an urgent problem.

As for the separation of ^177^Lu from irradiated Yb targets, the process takes a long time, and the loss of ^177^Lu can reach 15% [12]. At the same time, in the present work, we demonstrated the possibility of rapid and quantitative recovery of ^177^Lu from irradiated HfO_2_. When ^177^Lu is produced in a reactor, the long-lived ^177m^Lu isomer is completely absent [10]; when produced in an electron accelerator, isomer activity is 0.00287% of the activity of ^177^Lu; and during cyclotron production, isomer activity does not exceed 0.0045% of ^177^Lu one [11].

It is difficult to compare the cost of ^177^Lu obtained by different methods for a number of reasons. The cost of production in the reactor is the lowest, but this method has disadvantages mentioned in the Introduction, including radioactive waste generation. Per unit of time, a higher ^177^Lu activity is generated in the cyclotron than in an electron accelerator; however, the cost of the operation of the latter is lower. Finally, it is worth considering that the regeneration of HfO_2_ targets is easy, as we demonstrated, while ^176^Yb is usually not regenerated.

As a result, each of the described methods has its advantages and disadvantages, and each can be used to obtain ^177^Lu for nuclear medicine purposes. In any case, it is currently possible to produce ^177^Lu for preclinical studies in electron accelerators. Further development of the photonuclear method for obtaining ^177^Lu consists of establishing the exact values of the yields of the desirable isotope after irradiation of different enriched hafnium targets. 

## 3. Materials and Methods

### 3.1. Irradiation of HfO_2_

^nat^HfO_2_ with a weight of 16 g was placed in cylindrical polypropylene container with a volume of 5 mL; the remaining space in container was filled with cotton wool. The container was then irradiated for 8 h in RTM-55 microtron with maximum energy of electron beam being 55 MeV [14]. Tungsten plate of 2 mm thickness was used as an electron convertor; the usual value of average current was 100–200 nA for used accelerator. During radiochemical analysis of irradiated target isotopes ^177,178,179^Lu, ^173,175^Hf, ^89^Zr, and ^88^Y were found; the same isotopes were also observed in our work [8].

### 3.2. Target Dissolution, Recovery of ^177^Lu, and Regeneration of HfO_2_

Irradiated HfO_2_ was dissolved in HF_conc_ by boiling for 1.5 h. Obtained solution was diluted 15 times with 1 M HNO_3_, resulting in approximately 260 mL.

Four identical columns with volume of 3 mL and diameter of 0.6 cm each, filled with LN resin (100–150 mesh, Triskem Int, Bruz, France), were used in following recovery by extraction chromatography. The solution was divided into 4 equal portions; each was eluted through its own column. Fractions of 5 mL each were gathered during the elution; their gamma-ray spectra were registered using spectrometer with high-purity germanium detector Canberra GC1020 (Canberra Ind, Meridan, CT, USA). Content of hafnium, zirconium, and lutetium in fractions during recovery process was determined using gamma-peaks of the following isotopes: ^177^Lu (208.4 keV), ^175^Hf (343.4 keV), ^89^Zr (909 keV). Content of yttrium was determined during prolonged registration of spectra using ^88^Y peak (898 keV).

Regeneration of hafnium from the initial solution eluted through the column was conducted by adding ammonia to form precipitate of hafnium hydroxide. This precipitate was separated from the solution by filtration and then was heated for 4 h at 850 °C until the formation of HfO_2_. XRD spectra (Miniflex 600, Rigaku Corporation, Tokyo, Japan) of obtained product were compared to the spectra of initial HfO_2_ using database PDF-2.

Study of yttrium behavior on LN resin was carried out by determination of distribution coefficients using ^90^Y tracer. Content of ^90^Y in solutions was determined by liquid scintillation spectrometry (LS-spectrometer GreenStar, Moscow, Russia) using liquid scintillation cocktail UltimaGold (PerkinElmer Inc., Shelton, CT, USA), taking into account efficiency calibration for acid concentration.

### 3.3. Determination of Purification Degree of ^177^Lu and ^177m^Lu Content

1 mL was taken from fractions containing purified lutetium (80 mL) to determine its hafnium and zirconium content using quadrupole mass-spectrometer with inductively coupled plasma X-series II (Thermo Fisher Scientific, Dreieich, Hessen, Germany). Remaining lutetium solution was evaporated to dryness on a round steel plate with a diameter of 2 cm to determine content of ^177m^Lu and purification degree of ^177^Lu by radiometry. Activity of plate then was measured several times for the following 270 days using gamma-ray spectrometer with high-purity germanium detector GC3019 (Canberra Ind). Calibration of count efficiency depending on the energy of registered isotope was conducted using measurements of activity of certified point sources (^152^Eu, ^137^Cs, ^60^Co, ^241^Am) in different location geometries of source and detector and was also modeled in GEANT4. Identification of peak maximum in spectra was carried out using automatic system of spectrum record and analysis, specially created for this purpose. Thus, spectra with duration of 3.5 s each were saved into the database, and analysis system allowed us to summarize them and display total spectrum with assigned duration [15].

Purification degree of ^177^Lu from macroquantities of Hf and Zr was calculated by dividing the mass of Hf or Zr in the initial solution by the mass of ones in purified lutetium solution using ICP-MS data. Purification degree of ^177^Lu from microquantities of ^88^Y was determined by gamma-ray spectrometry.

## 4. Conclusions

A method of recovery of carrier-free ^177^Lu from macroquantities of hafnium and zirconium and trace amounts of yttrium was developed; the yield of lutetium was not less than 98%. Contents of hafnium, zirconium, and yttrium in the obtained solution of ^177^Lu were at least 1.19 × 10^10^, 1.7 × 10^6^, and 10^4^ times lower compared to the initial solution. The achieved level of ^177^Lu purification allowed us to determine the activity of ^177m^Lu during the prolonged registration of the gamma-ray spectrum, resulting in determination of the ^177m^Lu/^177^Lu activities ratio that reached a value of (2.87 ± 0.07) × 10^−5^ for the photonuclear method at the studied energy; this ratio indicates a high purity of the obtained ^177^Lu and the possibility of its use in nuclear medicine. The developed method was successfully applied to obtain ^177^Lu after the irradiation of 16 g of HfO_2_ in an electron accelerator. It was demonstrated that irradiated HfO_2_ could be quantitatively regenerated and later be reused for the production of the medical isotope ^177^Lu.

Thus, we demonstrate that it is possible to produce and quantitatively recover ^177^Lu for preclinical studies using an electron accelerator. Moreover, the photonuclear production of ^177^Lu can also become an alternative method for its obtaining, but to date, an experimental study of the yields of photoproton reactions on enriched targets made of ^178^Hf and ^179^Hf is required.

## Figures and Tables

**Table 1:** Quantities of ^177^Lu and other elements in eluate during different stages of ^177^Lu recovery.

Stage	Content of Elements in Eluate, % of Initial			
	Hf	Zr	Y	Lu
Sorption of ^177^Lu onto the column, rinsing with the mixture of 1 M HNO_3_ and 0.1 M HF	100	100	40	<2
Rinsing with 2.5 M HNO_3_	0	0	60	0
Desorption of ^177^Lu with 6 M HNO_3_	0	0	0	>98

**Table 2:** ^177m^Lu/^177^Lu activity ratios in production of ^177^Lu via different methods.

Nuclear Reactions Producing ^177^Lu	^177m^Lu/^177^Lu Activity Ratio
^178^Hf(γ,p)^177^Lu (determined in this work)	0.00287%
^178^Hf(γ,p)^177^Lu (determined previously)	<0.013% [8]
^176^Lu(n,γ)^177^Lu	<0.02% [9]
^176^Yb(n,γ)^177^Yb → ^177^Lu	0 [10]
^176^Yb(d,p)^177^Yb → ^177^Lu + ^176^Yb(d,n)^177^Lu	<0.0045% [11]

## Data Availability

Not applicable.

## Supplementary Materials

The following supporting information can be downloaded at: https://www.mdpi.com/article/10.3390/molecules27103179/s1, Figure S1: Distribution coefficients (Kd) of Y onto LN resin in HNO_3_, Figure S2: Elution curve of ^90^Y during elution of model solution containing 1 g of dissolved HfO_2_ through LN resin column.
