# Supplementary material for: Recovery of 177Lu from Irradiated HfO2 Targets for Nuclear Medicine Purposes
Source: Molecules. 2022 May 16;27(10):3179. doi: 10.3390/molecules27103179 (PMC9147805; doi:10.3390/molecules27103179)
Supplement: Supplementary file 1 [file molecules-27-03179-s001.zip › molecules-1682381-supplementary.pdf]

Supplementary.

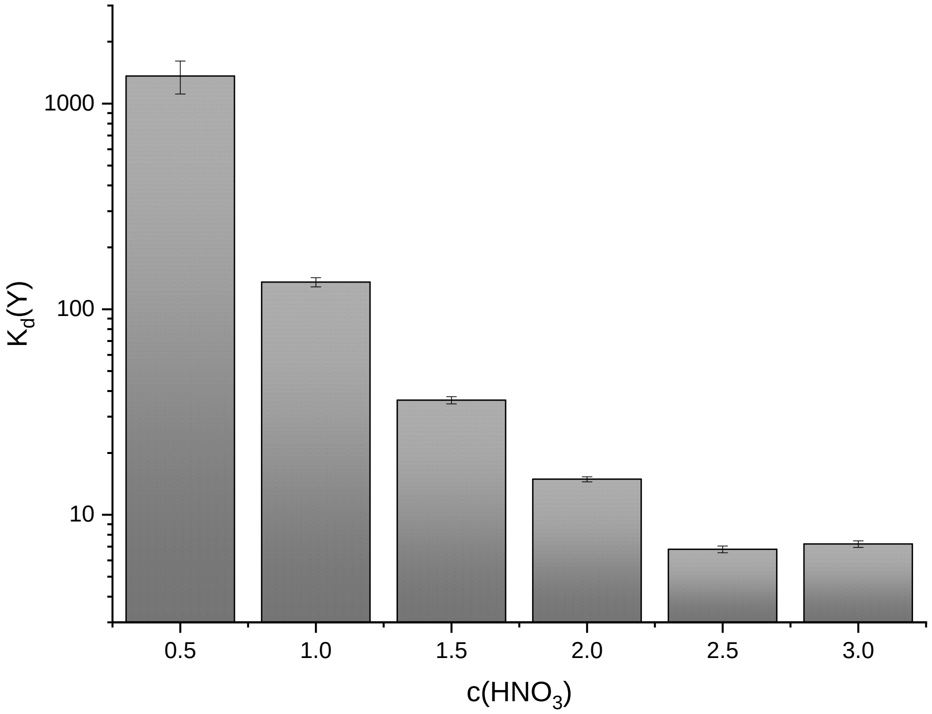

Figure S1. Distribution coefficients (K_d) of Y onto LN resin in HNO_3.

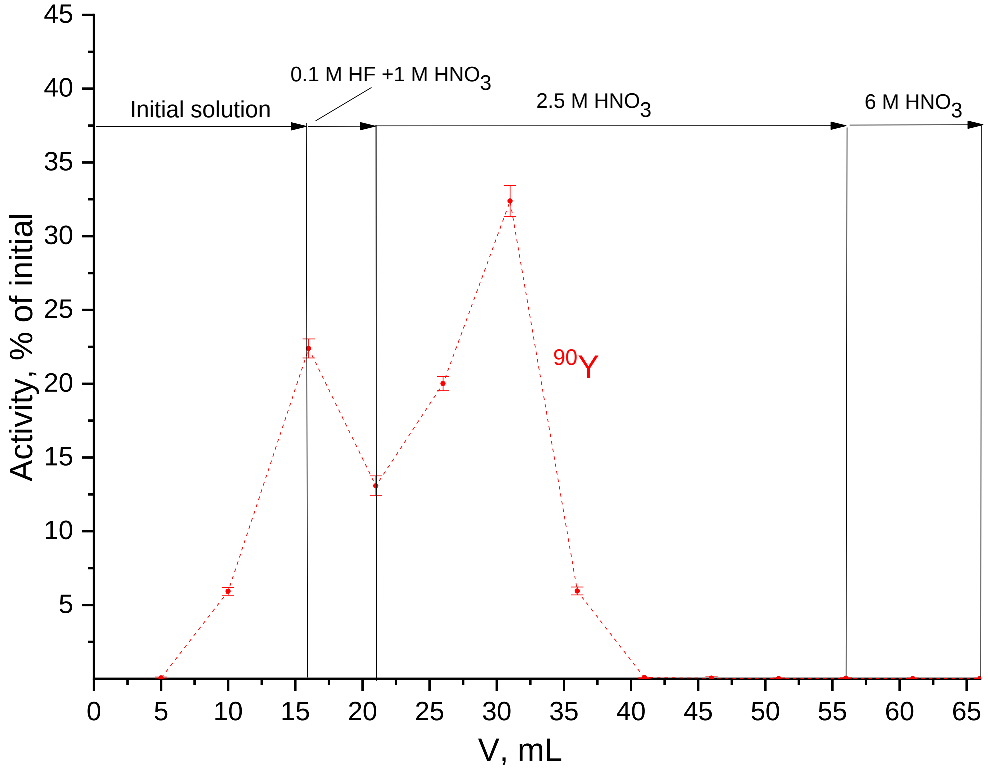

Figure S2. Elution curve of ^{90}Y during elution of model solution containing 1 g of dissolved HfO_2 through LN resin column.
